# Assessment of Normal Systolic Blood Pressure Maintenance with the Risk of Coronary Artery Calcification Progression in Asymptomatic Metabolically Healthy Korean Adults with Normal Weight, Overweight, and Obesity

**DOI:** 10.3390/jcm12113770

**Published:** 2023-05-31

**Authors:** Ki-Bum Won, Su-Yeon Choi, Eun Ju Chun, Sung Hak Park, Jidong Sung, Hae Ok Jung, Hyuk-Jae Chang

**Affiliations:** 1 Division of Cardiology, Dongguk University Ilsan Hospital, Dongguk University College of Medicine, Goyang 10326, Republic of Korea; kbwon99@naver.com; 2 Division of Cardiology, Severance Cardiovascular Hospital, Yonsei University College of Medicine, Yonsei University Health System, Seoul 03722, Republic of Korea; 3 Division of Cardiology, Healthcare System Gangnam Center, Seoul National University Hospital, Seoul 06236, Republic of Korea; sychoi9@gmail.com; 4 Division of Radiology, Seoul National University Bundang Hospital, Seongnam 13620, Republic of Korea; drejchun@hanmail.net; 5 Division of Radiology, Gangnam Heartscan Clinic, Seoul 06168, Republic of Korea; shparkmdmax@gmail.com; 6 Division of Cardiology, Heart Stroke & Vascular Institute, Samsung Medical Center, Seoul 06351, Republic of Korea; jidong.sung@gmail.com; 7 Division of Cardiology, Seoul St. Mary’s Hospital, College of Medicine, The Catholic University of Korea, Seoul 06591, Republic of Korea; hojheart@catholic.ac.kr

**Keywords:** systolic blood pressure, atherosclerosis, coronary artery calcium score, metabolically healthy obesity

## Abstract

Metabolically healthy obesity (MHO) is known to have a close association with subclinical coronary atherosclerosis. Despite recent data on the benefit of intensive systolic blood pressure (SBP) control in diverse clinical conditions, little is known regarding the association of normal SBP maintenance (SBP_maintain_) with coronary artery calcification (CAC) progression in MHO. This study included 2724 asymptomatic adults (48.8 ± 7.8 years; 77.9% men) who had no metabolic abnormalities except overweight and obesity. Participants with normal weight (44.2%), overweight (31.6%), and obesity (24.2%) were divided into two groups: normal SBP_maintain_ (follow-up SBP < 120 mm Hg) and ≥elevated SBP_maintain_ (follow-up SBP ≥ 120 mm Hg). CAC progression was defined using the SQRT method, a difference of ≥2.5 between the square root (√) of the baseline and follow-up coronary artery calcium score. During a mean follow-up of 3.4 years, the proportion of normal SBP_maintain_ (76.2%, 65.2%, and 59.1%) and the incidence of CAC progression (15.0%, 21.3%, and 23.5%) was different in participants with normal weight, overweight, and obesity (all *p* < 0.05, respectively). The incidence of CAC progression was lower in the normal SBP_maintain_ group than in the ≥elevated SBP_maintain_ group in only participants with obesity (20.8% vs. 27.4%, *p* = 0.048). In multiple logistic models, compared to participants with normal weight, those with obesity had a higher risk of CAC progression. Normal SBP_maintain_ was independently associated with the decreased risk of CAC progression in participants with obesity. MHO had a significant association with CAC progression. Normal SBP_maintain_ reduced the risk of CAC progression in asymptomatic adults with MHO.

## 1. Introduction

Obesity is a substantial cardiovascular (CV) risk factor [1,2], contributing to the development of CV disease through numerous metabolic abnormalities, including hypertension, hyperglycemia, and dyslipidemia [3,4]. It has been of interest whether individuals with obesity but without other metabolic abnormalities are still at an increased risk of CV disease [5,6]. For this population, the term of metabolically healthy obesity (MHO) has been used in clinical practice. A number of studies have reported that MHO is not a benign health condition with respect to atherosclerotic CV disease [7,8,9].

Recent randomized trials consistently reported the benefit of intensive systolic blood pressure (SBP) control for reducing major adverse events in patients with an increased CV risk [10,11]. Thus, the American College of Cardiology (ACC)/American Heart Association (AHA) guidelines lowered the blood pressure thresholds for the diagnosis of hypertension [12]. In addition, the Kidney Disease Improving Global Outcomes guidelines also recommend targeting SBP lower than or equal to 120 mmHg in chronic kidney disease patients excluding those with dialysis or a kidney transplant [13]. However, little is known as to whether the normal SBP maintenance (SBP_maintain_) is an effective strategy for attenuating coronary atherosclerosis in asymptomatic adult population with MHO. This is an important issue considering that recent data showed no significant association of body mass index (BMI) changes with the progression of coronary arteriosclerosis during a near-term period [14].

In asymptomatic adult population, the coronary artery calcium score (CACS) is widely used for CV risk stratification because of its prognostic significance beyond clinical risk factors [15,16,17]. In addition, coronary artery calcification (CAC) progression provides additional prognostic information in conditions without baseline heavy CAC [18]. This suggests that early detection of the presence and progression of CAC is important in the era of primary prevention. Numerous studies have suggested using CACS to determine therapeutic targets in various clinical conditions [19,20,21,22]. The present study aimed to evaluate the association between normal SBP_maintain_ and the risk of CAC progression among asymptomatic metabolically healthy Korean adults with normal weight, overweight, and obesity.

## 2. Materials and Methods

### 2.1. Study Design and Population

Data from the Korea Initiatives on Coronary Artery Calcification (KOICA) registry were analyzed in this study. The KOICA registry is a retrospective, single-ethnicity, multicenter, and observational study designed to investigate the effectiveness and prognostic value of CACS for primary prevention of CV disease in asymptomatic Korean adults [23,24]. All data were obtained using a health check database at the healthcare center of each site in South Korea (Severance Cardiovascular Hospital; Seoul National University Hospital; Seoul National University Bundang Hospital; Gangnam Heartscan Clinic; Samsung Medical Center; Seoul St. Mary’s Hospital). Initially, 93,914 Korean subjects who underwent a CAC scan that was performed at the time of voluntary visit to a healthcare center were enrolled in the KOICA registry between 2003 and 2017. For evaluating the changes of CAC, we identified 12,638 subjects who underwent at least two CAC scans among these subjects. After excluding 9914 subjects who had any component of metabolic syndrome (MetS) or incomplete data for identifying the component of MetS at enrollment, 2724 subjects were finally included in this analysis (Supplementary Figure S1). At each visit to a healthcare center, self-reported medical questionnaires were employed to collect information on the participants’ medical history. Height and weight were measured with the participants wearing light clothing and without shoes. BMI was calculated as weight (kg)/height (m^2^). Blood pressure was measured using an automatic manometer on the right arm after resting for at least 5 min. All blood tests, including total cholesterol, triglyceride, high-density lipoprotein (HDL) cholesterol, low-density lipoprotein (LDL) cholesterol, glucose, and creatinine, were obtained after at least 8 h of fasting. To measure CACS, computed tomography (CT) was performed with ≥16-slice multidetector scanners (Siemens 16-slice Sensation, Siemens, Forchheim, Germany; GE 64-slice Lightspeed, GE Healthcare, Milwaukee, WI, USA; Philips Brilliance 40-channel multidetector CT, Philips Healthcare, Cleveland, OH, USA; Philips Brilliance 256 iCT, Philips Healthcare). All scans were performed with a standard ECG-triggered scan protocol. The CACS was assessed by experienced CV radiologists, and the results were reported in the electronic health records. Informed consent for CT examination was obtained from each participant. All methods were performed in accordance with the relevant guidelines and regulations. The institutional review board committee of Yonsei University Health System, Severance Hospital approved the protocol of the present study (IRB No. 4-2014-0309).

### 2.2. Definitions

To define metabolically healthy condition except overweight and obesity, the present study employed the criteria of the National Cholesterol Education Program Adult Treatment Panel III for MetS as follows: (a) SBP ≥ 130 mm Hg or diastolic blood pressure (DBP) ≥ 85 mm Hg, previous history of hypertension, or the use of antihypertensive medications; (b) HDL cholesterol < 40 mg/dL in men or <50 mg/dL in women or the use of any medications for the treatment of dyslipidemia; (c) triglycerides ≥ 150 mg/dL or the use of any medications for dyslipidemia; and (d) fasting glucose ≥ 100 mg/dL, previous history of diabetes, or the use of antidiabetic medications [25]. Metabolically healthy condition except overweight and obesity was defined as clinical status without any components mentioned above. Categorical BMI was defined with the current Korean Society for the Study of Obesity Guidelines as follows: normal weight (18.5–22.9 kg/m^2^), overweight (23.0–24.9 kg/m^2^), and obesity (≥25.0 kg/m^2^) [26,27]. All participants with normal weight, overweight, and obesity were divided into two groups: normal SBP_maintain_ (follow-up SBP < 120 mm Hg) and ≥elevated SBP_maintain_ (follow-up SBP ≥ 120 mm Hg), respectively. Current smoking was defined as those who currently smoked or had smoked until 1 month before the study [28]. CACS was measured using the scoring system previously described by Agatston et al. [29]. CAC progression was defined using the SQRT method, as a difference of ≥2.5 between the square root (√) of the baseline and follow-up CACS (Δ√transformed CACS) [30,31], considering the proportion of CACS of 0 at baseline and the prognostic value of CAC progression defined using the SQRT method. Annual change of Δ√transformed CACS was defined as Δ√transformed CACS divided by the interscan period.

### 2.3. Statistical Analysis

The continuous variables are expressed as mean ± standard deviation. The categorical variables are presented as absolute values and proportions. After checking the distribution status of independent variables, we compared the participants’ characteristics among the categorical BMI groups using a one-way analysis of variance or a Kruskal–Wallis test for the continuous variables, as appropriate, and using the chi-squared test or Fisher’s exact test for the categorical variables, as appropriate. To compare the characteristics between normal SBP_maintain_ and ≥elevated SBP_maintain_, we employed an independent *t*-test or a Mann–Whitney U-test for the continuous variables, as appropriate, and the chi-squared test or Fisher’s exact test for the categorical variables, as appropriate. We used multiple logistic regression models to identify (1) the risk of CAC progression according to the categorical BMI and (2) the association of normal SBP_maintain_ with the risk of CAC progression in each categorical BMI. The forced entry method was used to enter independent variables for consecutive adjustment, including (1) unmodifiable clinical risk factors of age and sex, (2) modifiable clinical risk factors of SBP, DBP, and serum levels of triglyceride, HDL and LDL cholesterol, glucose, and creatinine, and current smoking, and (3) baseline CACS and interscan period into the multiple regression models. We performed all the statistical analyses using the Statistical Package for the Social Sciences version 19 (Chicago, IL, USA). A *p*-value of <0.05 was considered significant.

## 3. Results

### 3.1. Baseline Characteristics

The baseline characteristics of the overall population are shown in Table 1. The mean age was 48.8 ± 7.8 years, and 2122 (77.9%) were men. The proportion of participants with normal weight, overweight, and obesity was 44.2%, 31.6%, and 24.2%, respectively. The mean levels of SBP and DBP were 110.4 ± 10.3 mm Hg and 68.9 ± 7.9 mm Hg, respectively. Current smokers comprised 25.8% of the population.

Table 2 shows baseline CACS according to the categorical BMI. In participants with a higher BMI, the proportion with a CACS of 0 was decreased; in contrast, the proportion with CACS of 1–100 was increased. No significant difference in the proportion of participants with a CACS of >100 was observed among the participants. The mean values of CACS across three BMI groups were not significantly different (Supplementary Table S1).

### 3.2. CAC Changes according to Categorical BMI

**Figure 1:**
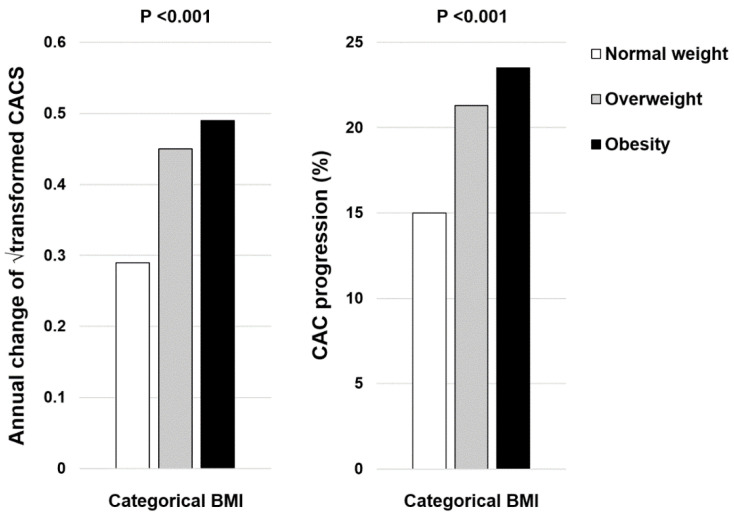
Changes of CAC according to categorical BMI.

During the mean follow-up of 3.4 ± 1.9 years, the annual change of Δ√transformed CACS (normal weight 0.29 ± 0.92 vs. overweight 0.45 ± 1.05 vs. obesity 0.50 ± 1.18, *p* < 0.001) and the incidence of CAC progression (normal weight 15.0% vs. overweight 21.3% vs. obesity 23.5%, *p* < 0.001) were increased in the participants with higher BMI (Figure 1). In the multiple logistic regression models, compared with participants with normal weight, those with obesity had a higher risk for CAC progression (Table 3).

### 3.3. Normal SBP_maintain_ and CAC Progression in Categorical BMI

**Figure 2:**
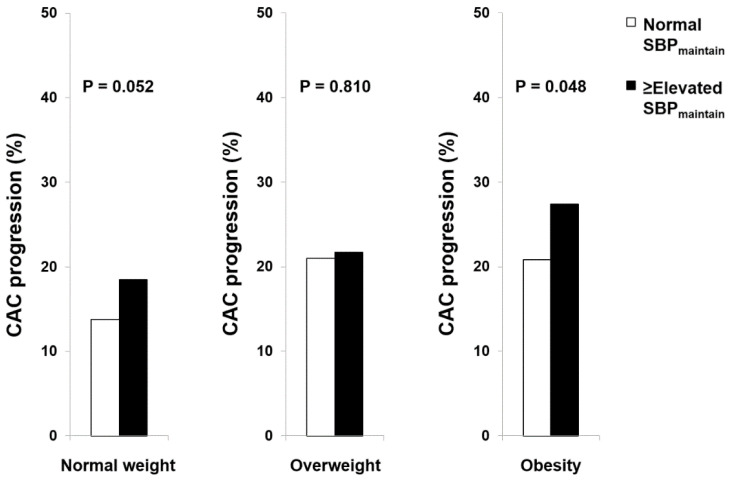
CAC progression related to normal SBP_maintain_ in categorical BMI.

The overall proportion of normal SBP_maintain_ was 68.6%; the proportion of normal SBP_maintain_ in participants with normal weight, overweight, and obesity was 76.2%, 65.2%, and 59.1%, respectively. At follow-up, the use of antihypertensive medication was identified in 4.6% of overall participants; the proportion of antihypertensive medication use in those with normal weight, overweight, and obesity was 2.8%, 6.0%, and 6.0%, respectively (*p* = 0.001). Compared with the ≥elevated SBP_maintain_ group, the incidence of CAC progression was not different in the normal SBP_maintain_ group among participants with normal weight (normal SBP_maintain_ 13.8% vs. ≥elevated SBP_maintain_ 18.5%, *p* = 0.052) and overweight (normal SBP_maintain_ 21.0% vs. ≥elevated SBP_maintain_ 21.7%, *p* = 0.810). However, the incidence of CAC progression was significantly lower in the normal SBP_maintain_ group than in the ≥elevated SBP_maintain_ group among the participants with obesity (20.8% vs. 27.4%, *p* = 0.048) (Figure 2). In multiple logistic regression models, normal SBP_maintain_ did not show a significant association with the risk of CAC progression among participants with normal weight and overweight. However, normal SBP_maintain_ was independently associated with a reduced risk of CAC progression in the participants with obesity (Table 4).

## 4. Discussion

To date, data on the change of subclinical coronary atherosclerosis related to normal SBP_maintain_ have been limited in asymptomatic metabolically healthy adults. Based on recent suggestions to use CACS for determining therapeutic targets in diverse clinical conditions, this study evaluated the association between normal SBP_maintain_ and CAC progression among asymptomatic metabolically healthy adults with normal weight, overweight, and obesity. In the present study, we initially confirmed that MHO was associated with the increased risk of CAC progression, as previous numerous studies suggested. The major finding of the present study is that normal SBP_maintain_ is associated with the decreased risk of CAC progression in conditions with MHO.

There has been no consensus on the definition of MHO [5]. In addition, the BMI cutoffs for defining obesity differ according to ethnicity. A previous cross-sectional cohort study by Chang et al. reported that MHO was associated with a higher prevalence of CAC than in metabolically healthy normal weight among 14,828 metabolically healthy Korean adults [7]. A recent longitudinal study from the Heinz Nixdorf Recall cohort found that subjects with MHO had a higher risk of CAC progression than metabolically healthy subjects with normal weight among 1585 Caucasian adults during a follow-up of 5 years [8]. Furthermore, Caleyachetty et al. identified that subjects with MHO had a higher risk of coronary heart disease, cerebrovascular disease, and heart failure than metabolically healthy subjects with normal weight in a Western population of 3.5 million adults without previous CV disease during a mean follow-up of 5.4 years [9]. Recent meta-analysis also showed that MHO was substantially associated with the increased risk of CAC [32]. These findings reveal that MHO is not a purely benign health condition regardless of MHO definitions and ethnicity.

The lowered blood pressure threshold for diagnosing hypertension by the 2017 ACC/AHA guidelines emphasizes the significance of blood pressure in preventing adverse CV events [12]. This change could lead to the overdiagnosis of hypertension and result in unnecessary treatment, particularly in young adults or in adults with low CV risk. Although both the SPRINT and STEP trials have recently shown the benefit of intensive SBP control in conditions with high CV risk [10,11], the significance of normal SBP_maintain_ for preventing the progression of subclinical coronary atherosclerosis has not been evaluated among metabolically healthy adults. In the present study, unlike in metabolically healthy adults with normal weight and overweight, normal SBP_maintain_ independently attenuated the risk of CAC progression in those with obesity. According to the data from the Progression of Atherosclerotic Plaque Determined by Computed Tomographic Angiography Imaging (PARADIGM) registry with a median follow-up of 3.3 years [14], the proportion of shift to a decreased BMI group was only 17.0% in obese subjects. It should be noted that that neither a categorical BMI group change nor a 5% change of BMI in the near-term period were associated with coronary plaque volume changes in the PARADIGM registry. Considering that the majority of subjects with MHO, about 59.1%, maintained normal SBP at follow-up in the present study, the normal SBP_maintain_ might be an effective target for preventing the progression of subclinical coronary atherosclerosis in conditions with MHO.

Among asymptomatic population, the CACS is an effective and noninvasive tool for CV risk stratification because of its powerful prognostic value across age, sex, ethnicities, and baseline risk factors [15,16,17]. Pharmacological agents such as statins could affect the progression of coronary atherosclerosis [33,34]. For this reason, coronary computed tomographic angiography (CCTA) has been proposed to have additional benefits over CACS and traditional risk factors in asymptomatic subjects due to its ability to provide more detailed information, including plaque burden and its composition. However, recent data from the CONFIRM (Coronary CT Angiography Evaluation For Clinical Outcomes: An International Multicenter) registry showed that further prognostic benefit was not conferred by CCTA when considering the traditional risk factors and CACS in an asymptomatic population [35]. Compared to the CV risk stratification using the traditional risk factors and CACS, the role of CCTA may be limited in asymptomatic clinical conditions.

There is a paucity of consensus for the definition of hypertension in recent clinical practice. Therefore, several studies have suggested the use of CACS to determine individualized therapeutic blood pressure goals [19,36], based on the recent evidence regarding the usefulness of CACS use in diverse clinical situations [21,37]. It may be possible that CACS helps to identify specific patients who may benefit from strict blood pressure control. Further outcome studies should be necessary to support this approach.

There are several limitations to the current study. First, the present cohort of self-referred asymptomatic subjects may not be fully representative of the general population. In addition, we only included participants without metabolic abnormalities except overweight and obesity at enrollment who underwent at least two CAC scans from the KOICA registry. Therefore, the characteristics of our study population did not represent the overall characteristics of the patients in the KOICA registry and a selection bias could be present. Second, different CT scanners were used among the participating centers. However, low inter- and intraobserver variability and high reproducibility across various vendors to measure CACS was well-established [38,39,40]. In addition, the same CT scanner was used with the ECG-trigger method at both image acquisitions. Third, because of the absence of a specific study protocol guiding follow-up CT scanning, the interscan period was relatively short and was not constant. However, we compared (a) the annual change of CAC and (b) the association of normal SBP_maintain_ with the risk of CAC progression after strict adjustment of confounding factors, including interscan period, among our participants with normal weight, overweight, and obesity. Fourth, data on consecutive changes in the clinical variables were unavailable during follow-up periods because of the observational nature of the study. In the present study, normal SBP_maintain_ was defined based on a follow-up SBP < 120 mm Hg without considering antihypertensive medication in participants with newly developed hypertension [41]. Some participants with normal SBP_maintain_ were misclassified into those with ≥elevated SBP_maintain_ because of white-coat effect and it could attenuate the effect ≥elevated SBP_maintain_ on the risk of CAC progression. However, this study identified the significance of normal SBP_maintain_ in subjects with MHO for preventing CAC progression despite the mentioned possibility. Fifth, this study did not evaluate the association of DBP status with the risk of CAC progression in the participants. Finally, considering that all the participants were Korean, it is hard to generalize the results of the present study. Despite these limitations, to the best of our knowledge, the present study is the first to identify that normal SBP_maintain_ is an effect target for reducing the risk of CAC progression in asymptomatic adults with MHO.

## 5. Conclusions

MHO was associated with a higher incidence of CAC progression among asymptomatic metabolically healthy Korean adults. Unlike in metabolically healthy subjects with normal weight and overweight, normal SBP_maintain_ independently reduced the risk of CAC progression in those with obesity. These results might imply that target levels of SBP for attenuating subclinical atherosclerosis might be somewhat different even in conditions with low CV risk. Further randomized investigations with larger sample size should be necessary to identify this issue.

## Figures and Tables

**Table 1:** Baseline characteristics.

Characteristics	*N* = 2724
Age, year	48.8 ± 7.8
Men, *n* (%)	2122 (77.9)
SBP, mmHg	110.4 ± 10.3
DBP, mmHg	68.9 ± 7.9
BMI, kg/m^2^	23.4 ± 2.4
Categorical BMI, *n* (%)	
Normal weight	1204 (44.2)
Overweight	860 (31.6)
Obesity	660 (24.2)
Current smoking, *n* (%)	702 (25.8)
Laboratory	
Total cholesterol, mg/dL	194.3 ± 31.4
Triglyceride, mg/dL	90.5 ± 28.5
HDL cholesterol, mg/dL	60.0 ± 16.1
LDL cholesterol, mg/dL	119.6 ± 30.8
Glucose, mg/dL	87.4 ± 7.1
Creatinine, mg/dL	0.9 ± 0.2

Values are presented as mean ± standard deviation or as number (%). BMI: body mass index; DBP: diastolic blood pressure; HDL: high-density lipoprotein; LDL: low-density lipoprotein; SBP: systolic blood pressure.

**Table 2:** Baseline CACS according to the categorical BMI.

	Normal Weight (*n* = 1204)	Overweight (*n* = 860)	Obesity (*n* = 660)	*p*
CACS	17.8 ± 88.7	16.4 ± 67.5	14.2 ± 54.9	0.596
Categorical CACS				
0	929 (77.2)	624 (72.6)	472 (71.5)	0.010
1–100	226 (18.8)	195 (22.6)	164 (24.9)	0.005
>100	49 (4.0)	41 (4.8)	24 (3.6)	0.532

Values are given as mean ± standard deviation or number (%). BMI: body mass index; CACS: coronary artery calcium score.

**Table 3:** Risk of CAC progression according to the categorical BMI.

	CAC Progression	
	OR (95% CI)	*p*
Model 1		
Normal weight	1	–
Overweight	1.29 (1.01–1.65)	0.040
Obesity	1.52 (1.17–1.97)	0.001
Model 2		
Normal weight	1	–
Overweight	1.21 (0.95–1.56)	0.129
Obesity	1.42 (1.08–1.84)	0.012
Model 3		
Normal weight	1	–
Overweight	1.14 (0.87–1.50)	0.331
Obesity	1.35 (1.01–1.82)	0.043

BMI: body mass index; CAC: coronary artery calcification; CACS: coronary artery calcium score; CI: confidence interval; DBP: diastolic blood pressure; HDL: high-density lipoprotein; LDL: low-density lipoprotein; OR: odds ratio; SBP: systolic blood pressure. Model 1: adjusted for age and sex. Model 2: Model 1 + adjusted for SBP, DBP, and serum levels of triglyceride, HDL and LDL cholesterol, and glucose. Model 3: Model 2 + serum creatinine levels, current smoking, baseline CACS, and interscan period.

**Table 4:** Association between normal SBP_maintain_ and CAC progression according to the categorical BMI.

	CAC Progression	
Categorical BMI	OR (95% CI)	*p*
Normal weight		
Model 1	0.85 (0.58–1.25)	0.414
Model 2	0.96 (0.63–1.45)	0.841
Model 3	0.99 (0.63–1.55)	0.957
Overweight		
Model 1	1.04 (0.73–1.49)	0.827
Model 2	1.04 (0.70–1.52)	0.860
Model 3	0.93 (0.61–1.42)	0.731
Obesity		
Model 1	0.64 (0.43–0.96)	0.029
Model 2	0.49 (0.31–0.78)	0.002
Model 3	0.55 (0.33–0.91)	0.020

*p* for interaction between categorical BMI and intensive SBP control was 0.919. BMI: body mass index; CAC: coronary artery calcification; CACS: coronary artery calcium score; CI: confidence interval; DBP: diastolic blood pressure; HDL: high-density lipoprotein; LDL: low-density lipoprotein; OR: odds ratio; SBP: systolic blood pressure. Model 1: adjusted for age and sex. Model 2: Model 1 + adjusted for SBP, DBP, and serum levels of triglyceride, HDL and LDL cholesterol, and glucose. Model 3: Model 2 + serum creatinine levels, current smoking, baseline CACS, and interscan period.

## Data Availability

The datasets used and analyzed during the current study are available from the corresponding author on reasonable request.

## Supplementary Materials

The following supporting information can be downloaded at: https://www.mdpi.com/article/10.3390/jcm12113770/s1, Figure S1: Flow chart of the study participant selection process; Table S1: Baseline CACS according to the categorical BMI.
