# Supplementary material for: Assessment of Normal Systolic Blood Pressure Maintenance with the Risk of Coronary Artery Calcification Progression in Asymptomatic Metabolically Healthy Korean Adults with Normal Weight, Overweight, and Obesity
Source: J Clin Med. 2023 May 31;12(11):3770. doi: 10.3390/jcm12113770 (PMC10253414; doi:10.3390/jcm12113770)
Supplement: Supplementary file 1 [file jcm-12-03770-s001.zip › [JCM] Supplementary table.pdf]

Supplementary Table S1. Baseline CACS according to the categorical BMI

	Normal weight (N = 1204)	Overweight (N = 860)	Obesity (N = 660)	P
Categorical CACS				
0	-	-	-	-
1–100	24.0 ± 25.5	21.0 ± 23.7	22.7 ± 24.5	0.471
>100	326.8 ± 300.5	243.3 ± 195.2	277.0 ± 243.7	0.164

Values are presented as mean ± standard deviation.
