# Supplementary figures and images for: Assessment of Normal Systolic Blood Pressure Maintenance with the Risk of Coronary Artery Calcification Progression in Asymptomatic Metabolically Healthy Korean Adults with Normal Weight, Overweight, and Obesity
Source: J Clin Med. 2023 May 31;12(11):3770. doi: 10.3390/jcm12113770 (PMC10253414; doi:10.3390/jcm12113770)

**Supplementary Figure S1.** Flow chart of the study participant selection process

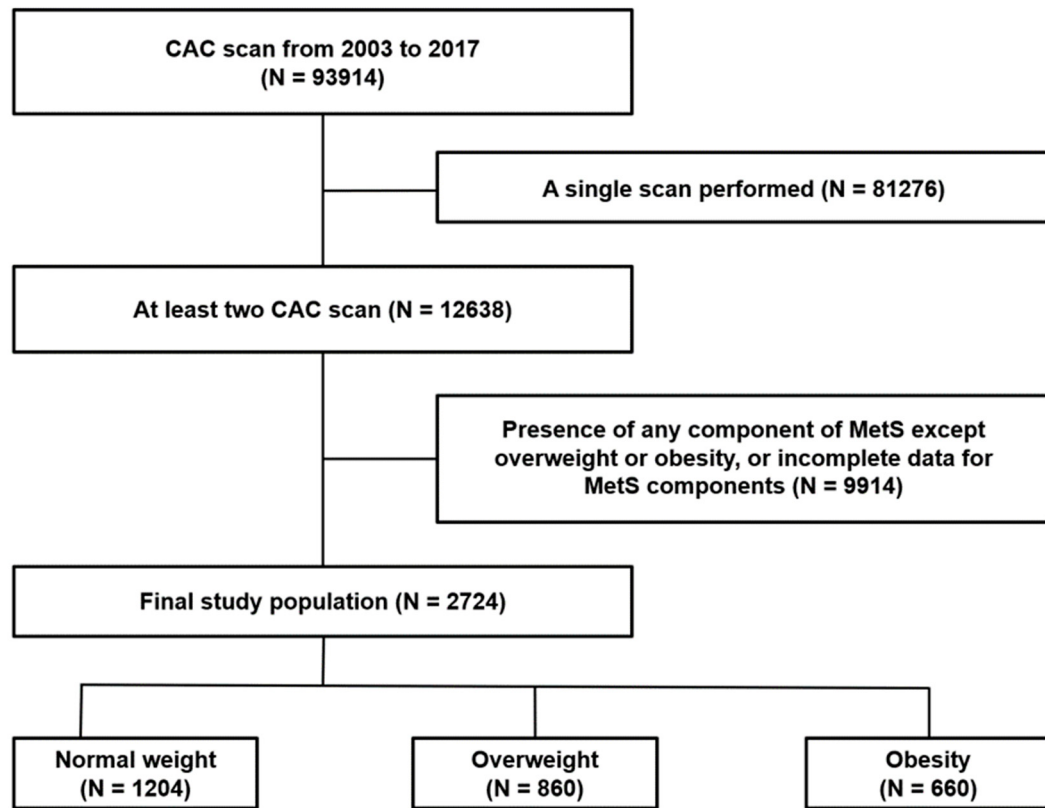

Supplement: Supplementary file 1 [file jcm-12-03770-s001.zip › [JCM] Supplementary figure.pdf]
